# Post-intensive care outpatient clinic: is it feasible and effective? A literature review

**DOI:** 10.5935/0103-507X.20180016

**Published:** 2018

**Authors:** Cassiano Teixeira, Regis Goulart Rosa

**Affiliations:** 1 Center for Adults Intensive Care, Hospital Moinhos de Vento- Porto Alegre (RS), Brazil.; 2 Postgraduate Program in Rehabilitation Sciences, Universidade Federal de Ciências da Saúde de Porto Alegre - Porto Alegre (RS), Brazil.

**Keywords:** Intensive care units, Patient discharge, Quality of health care, Ambulatory care

## Abstract

The follow-up of patients who are discharged from intensive care units follows distinct flows in different parts of the world. Outpatient clinics or post-intensive care clinics represent one of the forms of follow-up, with more than 20 years of experience in some countries. Qualitative studies that followed up patients in these outpatient clinics suggest more encouraging results than quantitative studies, demonstrating improvements in intermediate outcomes, such as patient and family satisfaction. More important results, such as mortality and improvement in the quality of life of patients and their families, have not yet been demonstrated. In addition, which patients should be indicated for these outpatient clinics? How long should they be followed up? Can we expect an improvement of clinical outcomes in these followed-up patients? Are outpatient clinics cost-effective? These are only some of the questions that arise from this form of follow-up of the survivors of intensive care units. This article aims to review all aspects relating to the organization and performance of post-intensive care outpatient clinics and to provide an overview of studies that evaluated clinical outcomes related to this practice.

## INTRODUCTION

The quantity^(1-3)^ and quality^(4-6)^ of life of patients who survive acute critical illness is a current concern of the intensivists and government authorities of certain countries of the world.^(7,8)^ The traditional and historical focus of intensive care has been on reducing mortality in the short term,^(9,10)^ but the survivors present significant mortality in the medium and long terms^(11-13)^ and can also experience a series of physical morbidities,^(6,14,15)^ cognitive dysfunction,^(16,17)^ depression^(18,19)^ and sexual dysfunction^(20,21)^ after discharge from the intensive care unit (ICU). In addition, post-discharge evolution of these patients presents with frequent hospital readmissions^(22)^ and with the use of many health resources,^(23)^ along with a high consumption of financial resources related to health.^(24)^

Follow-up outpatient clinics (or clinics) for ICU survivors were proposed as a way to follow up survivors after hospital discharge to treat the numerous morbidities prior to admission to the unit and to diagnose and treat those acquired during hospitalization in intensive care.^(25-28)^ In its conception, the main objective of the outpatient clinic was to improve the cost-effectiveness of care.^(25,29)^

In the UK, 30% of ICUs are undergoing outpatient follow-up,^(29)^ and current UK guidelines^(30)^ recommend that ICU survivors be reviewed 2 to 3 months after discharge. This evaluation focuses essentially on the diagnosis of motor and neuropsychological disorders to refer patients to specialized units when they are stricken by a problem (Table 1). This strategy serves to reduce the problems related to the fragmentation of the health system since a patient coming from an ICU is, by definition, a complex patient, morbid and with high short-term mortality risk.

**Table 1:** Objectives of post-intensive care unit outpatient clinics^(25,28,29,30)^

Goals	For patients	For family members and caregivers	For the ICU team
Diagnosis	Recognition of chronic diseases prior to ICU admission	Recognition of psychological changes acquired during the patient's stay in the ICU	Identification of physical and psychological sequelae of post-ICU discharge patients
	Recognition of chronic diseases acquired during ICU admission	Recognition of psychological changes acquired after the patient's stay in the ICU	Recognition of actions taken during ICU hospitalization that may have led to physical or psychological sequelae in patients and relatives
Counseling	Promotion of medication reconciliation	Guidance on psychological conditions related to patient care	Assistance from the ICU team in the understanding of post-ICU discharge sequelae
	Guidance on the prognosis of diseases acquired during ICU admission	Elucidation of doubts regarding the patient's stay in the ICU	Promotion of team well-being (reduction of burnout) by the recognition of their work by patients and their families
	Promotion of visits to the ICU to recall positive and negative passages that occurred during ICU admission		Promotion of meetings with patients and/or family members presenting "positive feedback" of patients and their family members regarding their stay in the ICU
	Elucidation of doubts regarding ICU admission		
	Promotion of better management of financial resources related to chronic conditions of patients in the health network		
Treatment	Meeting the functional rehabilitation needs of patients		
	Meeting the psychological rehabilitation needs of patients		

ICU - intensive care unit.

Basic concepts regarding the organization and efficiency of post-ICU outpatient clinic are described later in this article.

## ORGANIZATION OF POST-INTENSIVE CARE OUTPATIENT CLINIC

Post-ICU outpatient clinics vary widely in their need for professionals, patient and/or family eligibility for participation, time and duration of patient and family follow-up, choice of tools for evaluating outcomes, and definitions of which patients will be referred to reference services.^(30)^

Over the years, various follow-up strategies for patients from the ICU have been tested - some still in their very beginning and others with a body of evidence, not always favorable, but more robust, namely: (1) ICU team integration with primary public health;^(31,32)^ (2) peer support (i.e., formation of groups of patients who exchange experiences in face-to-face meetings or through internet sites);^(33)^ (3) frequent contacts of the ICU staff to address doubts through phone calls "telemedicine" programs;^(4)^ and (4) scheduled single or multiprofessional consultations.^(25,26,30)^

The need for professionals and the size of the action defined by them is very expensive for the health system. In this context, there is a strong participation of the financing model in the form of organization of post-ICU outpatient clinics.^(27,30)^ In the UK, most outpatient clinics are funded by the ICU itself, which allocates a preset monthly resource for monitoring these patients.^(25,30,32)^ In Brazil, these outpatient clinics are not part of the health policy strategies and, when they occur, are basically performed as clinical research stations in rare services.

### Eligibility of the professional

Depending on the model adopted and available resources, outpatient clinics can provide aid ranging from clinical to information services to ICU survivors and their families. Depending on the services available, the following may be offered: functional evaluation, physical therapy evaluation, medical evaluation, pharmaceutical evaluation, medical consultation, psychosocial support, and rehabilitation therapy, among others.^(27,28,30)^ The greater the range of professionals, the higher the cost of the operation.

In a study published in 2002 in the UK, outpatient clinics were primarily run by nurses or doctors.^(30)^ One-third of the outpatient clinics had access to psychotherapy services, and one-third had access to the physical therapy services. Specialized medical services were usually not routinely provided. In some outpatient clinics, a home physical therapy rehabilitation program was offered in addition to ambulatory patient appointments.^(34)^

### Eligibility of the patient

The type of patient referred to (or invited) to the post-ICU outpatient clinics varies according to the referred study (Table 2).^(20,26,31,34-60)^ However, most authors suggest that post-ICU outpatient follow-up should only occur in patients requiring mechanical ventilation ≥ 48 hours or ICU ≥ 2-5 days. It does not seem cost-effective to follow up all cases that have been discharged.^(28,34)^ Approximately 15-20% of patients admitted to the ICU meet these criteria, but less than 20% of these patients adhere to the program and are effectively followed up by outpatient services.^(28)^ This low attendance may possibly only mean that this model is not suitable for all patients. Our experience shows that the most dependent patients are unable to access the outpatient clinic due to the risk of not being transported safely.^(61)^ In our opinion, home visits could correct this problem, with results possibly similar to those in post-ICU outpatient clinics.

**Table 2:** Studies evaluating the follow-up of post-intensive care unit patients in outpatient clinics^(20,26,31,34-60)^

Study	County	Participant (n)	Clinical Staff	Inclusion criteria	Study type	Time of consultation (after ICU discharge)	Intervention or evaluation	Outcome or conclusion
Griffiths et al.^(20)^	United Kingdom	127	N/R	≥ 3 days of ICU	Observational	3, 6, and 12 months	Regular outpatient clinic for survivors of the ICU with application of specific questionnaires	43.7% reported symptoms of sexual dysfunction and there was relationship to PTS symptoms
Modrykamien et al.^(26)^	U.S.A.	N/R	N/R	N/R	Descriptive	N/R	Clinical follow-up and referral	N/R
Schmidt et al.^(31)^*	Germany	291 CG: 141 IG: 148	Nurse	Sepsis and septic shock	RCT	6 months	CG: primary care physicians IG: 12 months of regular contact with patients, reference to specialists, prescription of medications or other interventions. The intervention also consisted of training of primary care physicians and patients, management of cases provided by trained nurses, and clinical decision support (through specialists) for primary care physicians	There was no difference in the main outcome of the study, which was the change in quality of life related to mental health between discharge from the ICU and 6 months after discharge from the ICU, measured by the SF-36 Mental Component
Cuthberston et al.^(34)^*	Scotland	286 CG: 143 IG: 143	Doctor and nurse	Patients discharged from the ICU	Non-randomized CT	3 and 9 months	IG: self-monitored physical rehabilitation program started before hospital discharge, clinical evaluation by the nurse at 3, 9, and 12 months, discussion of ICU experiences, ICU visit, medication review, reference based on HADS score, cost analysis	192 patients completed 1-year follow-up There were no differences between the two groups regarding quality of life (EQ-5D), incidence of PTSD (Davidson Trauma Scale), depression and anxiety (HADS)
Lasiter et al.^(35)^*	U.S.A.	53	N/R	≥ 48 hours of MV or ≥ 48 hours of delirium	Descriptive	3 months	Evaluation by the interdisciplinary team (intensive care physician, nurse, social worker) and the creation of a personalized care plan, including cognitive exercises, self-management training manuals, pharmacological and non-pharmacological prescriptions, and proactive referrals to community resources, neuropsychologists, and physical rehabilitation services	Physical: patients who participated in 3 visits showed better physical performance in the 6-minute Walk Test and better leg strength over time Psychological: there were improvements in scores on anxiety, depression, and PTS scores
Jones et al.^(36)^*	United Kingdom	126	N/R	≥ 48 hours of ICU and patients in MV	RCT	8 weeks and 6 months	CG: received in-room visits, 3 home phone calls, and clinical consultations at 8 weeks and 6 months IG: received the same as the control group + 6-week self-help rehabilitation manual	There was improvement of the physical function (SF-36) in the intervention group, but the effect of the treatment may be related to the rehabilitation intervention, and not to the outpatient procedure per se
Engstrom et al.^(39)^	Sweden	9	Doctor and nurse	≥ 3 days of ICU and ≥ 24 hours of MV		6 months	Visit to ICU + debriefing about ICU stay + ICU diary review	The thematic analysis of these interviews revealed four fundamental roles of the post-ICU clinic: - ICU staff and family members reported that "they are given the strength to return together" - Patients found that experience allowed "to give meaning to the experience of critical illness" - Patients "felt grateful to have survived," and both survivors and family members appreciated the opportunity to meet with the ICU staff - Patients and family members viewed the visits as an "opportunity to improve care" and to return to the give a feedback to the ICU about their positive and negative experiences
Knowles et al.^(37)^*	United Kingdom	36CG: 18 IG: 18	Nurse	≥ 48 hours of ICU	Pragmatic RCT	2 months	IG: access to a prospective ICU diary kept by ICU nurses about events, treatments, procedures, and monitored conditions together with a verbal feedback from an ICU nurse in the psychological well-being, compared to a control condition without treatment	Prospective diaries designed to help patients understand what happened to them in the ICU significantly decreased anxiety and depression rates at the assessment performed 2 months after discharge from the ICU
Jones et al.^(38)^*	Europe	352 CG: 175 IG: 177	N/R	≥ 72 hours of ICU and ≥ 24 hours of MV	RCT	3 months	IG: patients received their prospective ICU diary in the first month after discharge from the ICU. A final evaluation of the development of acute PTSD was made during the 3-month period	The incidence of acute PTSD was significantly reduced in IG, especially in patients with higher scores
Crocker^(40)^	United Kingdom	6	Physician, nurse, physical therapist, and occupational therapist	≥ 4 days of ICU	Description of cases	2, 6, and 12 months	Visit to ICU + referral to specialist = drug reconciliation + physical therapy and occupational therapy assistance	Description of the experience of a multidisciplinary clinic
Hall-Smith et al.^(41)^	United Kingdom	26	Nurse	≥ 5 days of ICU	Unstructured interviews conducted by clients	Room, 2, and 6 months	Clinical interview	Description of the neuropsychological and physical findings of patients
Granja et al.^(42)^	Portugal	29	N/R	ARDS	Paired prospective cohort (patients without ARDS)	6 months	Evaluation in the post-ICU outpatient clinic	The quality of life of patients with ARDS was similar to that of other critically ill patients
Fletcher et al.^(43)^	United Kingdom	22	N/R	≥ 28 days of ICU	Prospective Cohort	N/R	After consultation with a general practitioner, all patients were invited to follow-up with the post-ICU outpatient clinic	Evaluation of the incidence of muscular weakness through electromyography
Kvale et al.^(44)^	Norway	346	Physicians	≥ 24 hours of ICU	Prospective Cohort	7 - 8 months	Respond to a survey in the ICU post-discharge and refer to an expert	Reduction of the quality of life (SF-36) in most patients
Flatten^(45)^	Norway	N/R	N/R	N/R	Editorial and descriptive population statistics	N/R	Regular outpatient clinic for ICU survivors	
Sukantarat et al.^(46)^	United Kingdom	51	N/R	≥ 3 days of ICU	Prospective, descriptive and correlational	3 and 9 months	Patients were recruited at a follow-up clinic at 3 and 9 months. No report on the clinic was included. The psychologist discussed the results of the research	45 patients completed the study Large proportion of patients with symptoms of anxiety, depression, and PTS
Holmes et al.^(47)^	Australia	90 CG: 39 IG: 51	Physician	Polytrauma with ≥ 24 hours of MV	RCT	3 and 6 months	CG: Interpersonal counseling with trained psychiatrist	77 patients completed the study The intervention was not effective to reduce psychiatric morbidity after a physical trauma, which can increase morbidity in vulnerable individuals
Douglas et al.^(48)^	U.S.A.	335 CG: 103 IG: 231	Nurse	≥ 3 days of MV	Near-experiment	2 months	Intervention centered on case management and interdisciplinary communication	247 patients completed the study There was no difference in patients' quality of life (SF-8)
Samuelson et al.^(49)^*	Sweden	170	Nurse	≥ 48 hours of ICU	Descriptive and evaluative	2 - 3 months	Visits in the ward (1-3 days after discharge from the ICU) + information flyer to patient + offer of a nurse telephone number for post-service + follow-up letter to provide information and offer a follow-up visit 2 - 3 months after discharge from ICU Patients' diaries with photographs were delivered. Long-term health rehabilitation counseling was provided, including identifying existing problems. A visit to the ICU, if they wished.	82% of factual and delusional ICU memories 51% remembered the visit of the post-care ward 60% remembered the information pamphlet. Those who remembered evaluated the experience of the ward visit between 9.3 and 9.7 (out of 10). The 2-month follow-up visit achieved a median score by patients and family members Some patients described in detail how information, explanations, and support enabled them to complete the puzzle of ICU stay and helped them move forward
Schandl et al.^(50)^*	Sweden	61	Physical therapist, pain clinician, and psychiatrist	≥ 4 days of ICU	Descriptive	3, 6, and 12 months	Visit to the ward + ICU diary + offer of follow-up at the clinic at 3, 6, and 12 months after discharge from the ICU	Multidisciplinary follow-up was able to identify untreated physical and psychological problems
Glimelius Peterson et al.^(51)^	Sweden	96	Physician and nurse	≥72 h of ICU	Exploratory	Immediate discharge, 2 and 6 months	In-room and clinic visit + outpatient or telephone follow-up	Reported as important by patients to elucidate doubts
Dettling-Ihnenfeldt et al.^(52)^	Netherlands	65	N/R	≥ 48 hours of VM	Prospective cohort	3 months	Comparison of 2 post-ICU outpatient clinics models (evaluation by SF-36 and HADS)	Most patients had significant functional restrictions
Jensen et al.^(53)^*	Denmark	386 CG: 196 IG: 190	Nurse	≥ 48 hours of MV	RCT	1 - 3, 5, and 10 months	IG: recovery program based on theoretical approaches to psychological recovery, including Antonovsky's salutogenic model, disease narratives, person-centered communication, elements of guided self-determination, and cognitive-behavioral therapy focused on trauma	There was no difference in quality of life, risk of anxiety and depression, and sense of coherence
Daffurn et al.^(54)^	Australia	54	Physician and nurse	≥ 48 hours of ICU	Prospective cohort	3 months	Semi-structured interview + clinical examination + ICU visit + referral to medical specialists or other health professional	Patients presented mild-moderate physical and psychosocial sequelae, but these symptoms did not impede their activities of daily living
Waldmann^(55)^	United Kingdom	N/R	Physician and nurse	≥ 4 days of ICU	Theoretical with descriptive statistics	2, 6, and 12 months	ICU visit + specialist referral + tracheostomy management + pulmonary function tests	N/R
Eddleston et al.^(56)^	United Kingdom	143	N/R	Patients discharged from the ICU	Prospective cohort	3 months	Visit the clinic in the third month for evaluation	Description of the findings referring to patients' quality of life
Sharland^(57)^	United Kingdom	N/R	N/R	≥ 4 days of ICU or referenced by ICU staff	N/R	2, 6, and 12 months	ICU visit + interview + information on rehabilitation + reference to specialists	N/R
Cutler et al.^(58)^	United Kingdom	N/R	Nurse	≥ 5 days of ICU	N/R	6 months	ICU Visit after discharge	N/R
Combe^(59)^*	United Kingdom	35	N/R	≥ 4 days of ICU	Prospective cohort	2, 6, and 12 months	Patients received their ICU diary at the first consultation (2 months) at the clinic with a later informal meeting	There was a better understanding of ICU events by the patients and improved communication with their family members
Jones et al.^(60)^*	United Kingdom	39	Nurse	Patients discharged from the ICU	Prospective audit	N/R	Nursing counseling	Patients required fewer counseling sessions. There was no difference in psychological outcome profiles

N/R - not reported; ICU - intensive care unit; PTS - posttraumatic stress; CG - control group; IG - intervention group; RCT - randomized clinical trial; SF-36 - 36-Item Short Form Health Survey; CT - clinical trial; HADS - Hospital Anxiety and Depression Scale; EQ-5D - Euro Quality of Life 5 Dimensions; PTSD - posttraumatic stress disorder; MV - mechanical ventilation; ARDS - acute respiratory distress syndrome.

* Those in which interventions were performed.

### Family member eligibility

Some experts suggest that caregivers and family members of ambulatory patients are also evaluated because of the high frequency of psychological disorders found in these individuals.^(18,25,27,30)^ Some authors also recommend that relatives of patients who died in the ICU could benefit from outpatient follow-up.^(28)^

### When to start and for how long to keep outpatient follow-up?

Post-ICU outpatient follow-up studies vary greatly regarding time of initiation of follow-up (Table 2). Van der Schaaf et al.^(27)^ suggest that the first visit to the outpatient clinic should be performed between the 6th and 12th week post-discharge. This time seems to be appropriate for patients, families, and caregivers to understand that motor sequelae may not heal as quickly as expected, and so they understand that changes in household architecture, structure, and family organization will be needed for a long period of time. In our opinion, a very late onset of outpatient follow-up (> 6 months after hospital discharge) is aimed only at the diagnosis of the functional and cognitive sequelae of patients and at the verification of family psychological and organizational problems already installed due to the lack of previous guidelines by the health team. Furthermore, missing the possibility of guiding these patients and their family members/caregivers would be very large, since approximately 25% of patients who were discharged from the ICU are either readmitted or die in the first months after discharge from the ICU.^(8,9,11,13,15)^

We are not aware of recommendations on how long patients, family members, and caregivers should remain in follow-up in post-ICU outpatient clinics. Data from the literature suggest that up to 10 years after hospital discharge, patients still present problems related to ICU admission,^(1,14)^ and that up to 1 year after hospital discharge, family members and caregivers present psychological and psychiatric symptoms related to the psychological load suffered in the ICU.^(62)^

### Evaluation instruments

The literature does not provide information to define which instruments should be used in the evaluation of outpatients after ICU discharge.^(27)^ However, most authors agree that a structured collection based on parameterized manual or electronic instruments can facilitate the interpretation of data *a posteriori* and can reduce costs related to personnel costs.^(27)^

## MEASUREMENT AND ACTION IN OUTCOMES

### Outcomes for patients

As shown in table 2, the evidence from the qualitative studies, as opposed to the quantitative ones, had a positive impact on the experience of the patient and the family members who participated in the post-ICU outpatient clinics.^(30)^ This difference in the findings of the quantitative studies, in relation to the qualitative ones, is likely due to the need: (1) to apply, in quantitative studies, instruments not validated for this population (e.g., 36-Item Short Form Health Survey - SF-36 and EQ-5D), (2) to self-complete many of the questionnaires (in patients with cognitive dysfunction); and (3) that patients exaggerate to please health care providers in qualitative interviews (e.g., patients exaggerate the benefit achieved by the intervention). In our view, rather than classifying these results as discordant, we consider them complementary, since qualitative studies should be viewed as hypotheses that generate and provide detailed information on survivors' experience, while quantitative ones try to translate these experiences into graphical or measurable forms.^(30)^

In the follow-up of patients discharged from the ICU, some clinical outcomes are very relevant and very prevalent. These patients often experience physical morbidities (functional decline, loss of ability to perform Daily Life Activities, and pain),^(6,14,15)^ cognitive dysfunction,^(16,17)^ depression,^(18,19)^ anxiety, posttraumatic stress disorder (PTSD), and sexual dysfunction.^(20,21)^ Objectively, up to the present time, much was measured, but little was obtained regarding the reduction of post-ICU discharge sequelae with actions performed in post-ICU outpatient clinics (Table 3).^(35-38)^ The most encouraging study is the meta-analysis of Jensen et al.,^(63)^ evaluating five studies that demonstrated protective effects on PTSD risk between the third and sixth months after discharge from the ICU (hazard ratio: 0.49, 95% confidence interval - 95% CI: 0.26 - 0.95) in patients undergoing follow-up by outpatient clinic teams; however, there were no reductions in the other domains of patients' quality of life.

**Table 3:** Quantitative studies demonstrating improvement of patient outcomes by means of actions related to the post-intensive care unit outpatient clinics^(35-38)^

Estimated outcome	Result
PTSD	Reduction of the risk of PTSD (0.49, 95% CI: 0.26-0.95) in 3-6 months after discharge from the ICU^(36)^ Improvement in PTSD scores in the third month after discharge from the ICU^(35)^ Reduction of acute PTSD in the third month of evaluation after discharge from the ICU^(38)^
Cognitive decline	-----
Anxiety	Improvement in anxiety scores in the third month after discharge from the ICU^(35)^ Reduction of anxiety symptoms assessed 2 months after discharge from the ICU^(37)^
Depression	Improvement in depression scores in the third month after discharge from the ICU^(35)^ Reduction of symptoms of depression assessed 2 months after discharge from the ICU(37)
Sexual disturbance	-----
Functionality/ADL	Better performance in the WT-6 evaluated in the third month after discharge from the ICU^(35)^ Improvement of physical function assessed by the SF-36 in 2-6 months after discharge from the ICU^(36)^
Hospital readmission	-----
Post-ICU mortality	-----

PTSD - posttraumatic stress disorder; ICU - intensive care unit; ADL - Activities of Daily Living; WT-6 - 6-Minute Walk Test; SF-36 - 36-Item Short Form Health Survey.

### Outcomes for family members and caregivers

Not only does an ICU stay have a lasting impact on the survivors, but it can also profoundly affect the family members and/or caregivers of these survivors.^(62)^ Since the primary focus of post-ICU outpatient clinics is on the patient, few outpatient follow-up studies have focused on the needs and morbidities of family members or caregivers. Although family members have often been encouraged to participate in clinical consultations in these rare reports, efforts have not always been specifically directed at the diagnosis or management of the patient's symptoms.^(30)^ Still, family members can subjectively report positive experiences with follow-up clinics,^(30)^ but many of them do not show up for follow-up at outpatient clinics.^(34)^

In the PRaCTICaL study,^(34)^ though the family members were invited to participate in the outpatient clinic, only one-third of them actually opted for the service. Engstrom et al.^(39)^ included nine family members in the outpatient evaluation, who reported that it had been a positive experience. In a randomized clinical trial, Jones et al.^(64)^ analyzed the psychological morbidity in caregivers before and after clinical intervention (ICU journal and rehabilitation). They documented PTSD symptoms in 49% of caregivers, anxiety symptoms in 58 - 62%, and depression in 22 - 31%, but found no effects of the intervention on any of these symptoms. These authors demonstrated that the family members reported that the experience of outpatient care was positive (subjective assessment). In this study, however, both groups participated in the clinical follow-up, with the addition of a manual and a rehabilitation program in the intervention group. Since the intervention was not directed specifically to caregivers, it is difficult to assess whether the intervention would have an impact on the psychological morbidity of the caregiver.

### Outcomes for the intensive care unit team

The experience of the ICU workers in caring for critically ill patients and their families leads to high levels of burnout*,* a well-documented phenomenon among ICU teams.^(65)^ Some authors argue that the professionals can suffer less burnout if they feel their work is valued and important to others.^(30)^ In analyzing narrative interviews^(66)^ with intensive care nurses, the researchers demonstrated that nurses appreciated the experience of "seeing a healthy person." In addition, they described that when patients and family members visited the ICU after discharge, this visit was "a learning experience." Currently, to our knowledge, there are no studies assessing burnout among ICU staff members participating in post-ICU outpatient clinics.

## POSSIBLE INTERVENTIONS TO BE CAPTAINED BY AN OUTPATIENT TEAM DEDICATED TO THE POST-DISCHARGE FROM THE INTENSIVE CARE UNIT

There are numerous possible interventions to be performed on patients, family members, and caregivers that can be captained by a team dedicated to post-ICU care. Briefly, some were described below.

### Integration with primary care (family doctor)

In the flow of the health system patients (health centers, emergency units, and hospital emergency departments), relevant information related to their health may be lost in a fragmented and inefficient care transfer model (*handover* PRaCTICaL*)*.^(67)^ Patients with diseases that do not show signs of severity should be treated in the primary care network (primary system) and should be referred to referral services (of greater complexity) when required. The ICU is situated at the apex of the complexity pyramid of this system. The need for technological resources and the high performance of professionals has led to a reduction in the mortality of patients in the intensive care setting.^(9)^

When they survive, these patients are referred to patient rooms (or wards) and later to their homes, featuring a progressive escalation of the need for care and, theoretically, the need for vigilance. However, these survivors frequently present new needs (e.g., tracheostomy, dialysis therapy, gastrostomy, and ventilatory support, among others), and their family members and basic health care staff may not be prepared for their proper care. Therefore, there is a risk that primary care physicians will be excluded from the clinical discussions and management of these ICU survivors.^(27,32)^ Family doctors may feel unqualified to manage and coordinate their complex and sometimes specialized needs, such as tracheostomy care, vocal fold dysfunction, muscle weakness, and PTSD, among others. However, should the intensivist doctors, who are scarce everywhere in the world,^(68)^ not remain in the ICU? In addition, intensive care physicians do not usually have health relationships with patients' family members or even communicate with family physicians, who are generally familiar with the overall situation of the patient and his/her family. In our opinion, it seems essential that intensivist doctors and family physicians work closely together in the management of these patients.

Schmidt et al.^(31)^ randomized 291 sepsis survivors to be maintained in the usual post-ICU care with their primary care physicians (n = 143) or to undergo intervention (n = 148) in nine ICUs in Germany, which consisted of 12 months of periodic outpatient contact with patients (by trained nurses), training of primary care physicians and patients in specific situations, support for clinical decision (by specialists) for primary care physicians, reference to specialists when needed, and prescription of medications when necessary. The main outcome of the study was the change in quality of life related to mental health on immediate discharge from the ICU and 6 months after discharge from the ICU, measured using the SF-36 Mental Component. The authors concluded that among survivors of sepsis and septic shock, the use of a team-based intervention focused on primary care compared with usual care did not improve the quality of life related to mental health 6 months after discharge from the ICU.

### Peer support

Issues regarding "staying alive" are rarely addressed by ICU teams during the time the patient is admitted to intensive care.^(69)^ Because the translation of knowledge is notoriously slow, many family physicians may not know of the existence of post-intensive care syndrome, rendering them even less likely to address survival issues. The result is that millions of critical illness survivors are discharged into the community, unprepared and uninstructed about what to expect from their recovery and how best to cope, adjust, and optimize their possible recovery. Since the patient's motor and neuropsychological impairments are often not recognized and/or minimized, a substantial burden usually falls on informal caregivers and family members - many of which can still be fighting their own emotional sequelae secondary to the patient's experience in the ICU.^(70)^

Peer support is a strategy in which patients help patients and can be defined as "a process of empathy, which offers advice and share stories among ICU survivors".^(33)^ The mutual support is based on mutual respect. Peer support is centered on the notion that survivors can help each other, share problems, and the will to overcome them. It is not a physician-centered model; however, it has the role of helping to provide a safe space in which survivors can work.^(33)^ The potential benefits of this technique come from establishing a community that promotes health and well-being through shared experiences of disease and recovery. Potential benefits include mental reassignment (hope and optimism), role modeling, sharing of information, and practical advice that is not readily available to health professionals.^(33)^ In addition, peer support has already proven to be an effective technique in people with mental health disorders and substance abuse problems, in the self-management of diabetes, and among cancer survivors.^(33)^

As survivors and their caregivers have firsthand experience with the challenges they will face, these individuals are well-suited to educating and preparing other survivors for certain aspects of the recovery process. However, spirituality and religion seem to be very important in survivor support networks,^(71)^ and because of the reluctance of health care providers to engage in the spiritual aspects of illness and recovery,^(72)^ these support groups can allow patients to explore these aspects of recovery with greater fluidity.

### Monitoring of patients by telephone or telemedicine

A large part of the follow-up studies of ICU survivors was performed through telephone contacts and the application of validated and standardized questionnaires and instruments.^(4)^ This form of screening can improve resources by detecting only those patients at higher risk of developing physical and neuropsychological disorders, that is, possible candidates for post-ICU outpatient follow-up.

The use of telemedicine in the follow-up of patients from the ICU can be an instrument that facilitates communication between family physicians and critical care medical and non-medical specialists. In the future, this tool can also provide real-time decision making for ICU survivors in any region of the country, bringing ICUs to cities other than capitals and making intensive care more balanced in different regions of Brazil.

### Reconciliation of medicines

A review of which medications will be required after ICU discharge is a fundamental stage of post-ICU care often neglected by the teams of these units.^(25,30)^ These patients are characteristically at high risk when compared with other hospitalized patients; frequently, their continued use medications are discontinued during hospitalization and are not - either intentionally or unintentionally - restarted after discharge from the ICU. The return of the patient to the post-ICU outpatient clinic can be an excellent opportunity to review the medication in use, aiming at restarting, maintaining, or withdrawing drugs.^(30,40)^

### Provide palliative care

Currently, palliative care specialists encourage a broader view of their specialty, with a focus on symptom relief and the promotion of quality of life for individuals with chronic but lifelong illnesses.^(32)^ Consultation in palliative medicine in the context of post-ICU outpatient clinics translates the use of an excellent opportunity to meet the patient's many needs, such as psychological concerns, spiritual needs, and better physician-patient, physician-family, and family-patient communication.^(32,41)^

### Referrals for specialties

Undoubtedly, one of the great contributions that outpatient clinics can give patients and their families is through standardized assessments to confirm psychological, cognitive, or functional diagnoses that allow them to be referred for specific evaluations and treatments in the health care network.

## CHALLENGES IN THE FOLLOW-UP OF PATIENTS POST-DISCHARGE FROM THE INTENSIVE CARE UNIT

Currently, there is scant evidence as to which is the best follow-up model for ICU survivors.^(30,31,42)^ What is the most cost-effective model? Which model is able to provide the service equitably to patients? Does regionalization of the UTI, more preferably concentrated in large cities, allow a single model of post-ICU care? Is a single model of post-ICU care appropriate for functionally dependent and independent patients? Which professionals are the most qualified to implement each model of post-ICU care? Should the post-ICU outpatient clinic be uni- or multidisciplinary?

Table 4 describes the authors' opinions with respect to organizational aspects and outcomes to be measured in the post-ICU outpatient clinic.

**Table 4:** Structure of the post-intensive care unit outpatient clinic target to patients only and not to family members and/or caregivers (authors' suggestion)

Post-ICU Stages	Who evaluates?	Who is evaluated?	How?	When?
Immediate post-discharge	Intensivist nurse	Patients requiring ICU ≥ 3 days	Face-to-face assessment of the degree of dependence (e.g., Barthel's Modified Scale)	During the first week after discharge from the ICU (still in the hospital)
Screening	Intensivist nurse	Patients assessed in the immediate post-discharge	Telephone evaluation on the degree of dependence (e.g., Barthel's Modified Scale) and on anxiety/depression symptoms (e.g., HADS) and PTSD (e.g., IES)	1-2 months after ICU discharge
Outpatient evaluation	Intensivist nurse and intensive care physician	Patients who present alterations in some of the questionnaires performed during screening	Face-to-face assessment of the degree of dependence (e.g., Barthel's Modified Scale) and cognition (e.g., MEEM)	3 months after discharge from ICU
Telephone evaluation	Intensivist nurse	Patients who were in the outpatient clinic consultation	Telephone assessment on the degree of dependence (e.g., Barthel's Modified Scale) and on anxiety/depression symptoms (e.g., HADS) and PTSD (e.g., IES)	12 months after discharge from ICU

ICU - intensive care unit; HADS - Hospital Anxiety and Depression Scale; PTSD - posttraumatic stress disorder; IES - Impact Event Scale; MEEM - Mini-Exam of the Mental State.

## FINAL COMMENTS

In this review, the importance of the ICU team in building and maintaining long-term relationships with their patients has been emphasized. Some authors suggest that ICU staff should continue to follow patients after they leave the ICU and hospital, especially those for whom we indicate and perform high-complexity and high-cost interventions.^(43)^

To date, the model of outpatient follow-up after ICU discharge does not seem to provide significant benefits to patients and families and is not cost-effective. However, this limitation should not reduce the importance of the long-term follow-up of patients, family members, and caregivers. Patients feel confident with the participation of intensivists in their future therapeutic decisions since the complexity of their sequelae requires multidisciplinary and specialized follow-up. Relatives feel confident in clarifying their doubts and exposing their fears to the team that treated them with respect and dignity during what was possibly the worst situation in their lives.

Therefore, our challenge is to develop and implement longitudinal models of care that begin the day the patient enters the ICU and continue for the rest of the hospitalization, and even after it. Our focus should start with the prevention of morbidity, early initiation of rehabilitation activities, and management of delirium, attitudes known to impact the long-term results.^(44)^ The use of an events diary kept by the ICU staff and presented to patients after discharge seems to have benefits in reducing the symptoms of PTSD.^(42,63)^ Some colleagues have argued for a follow-up model that prioritizes the best use of technology, such as telemedicine and electronic health records, aimed at better communication with primary care providers, rehabilitation institutions, and experts responsible for specialized clinical assessments.^(35)^ Perhaps, and most likely, there is no single model of monitoring of post-ICU patients, but several models which, when individualized, allow the "right care for the right patient".
